# Mechanochemical Activation and Spark Plasma Sintering of the Lead-Free Ba(Fe_1/2_Nb_1/2_)O_3_ Ceramics

**DOI:** 10.3390/ma14092254

**Published:** 2021-04-27

**Authors:** Dariusz Bochenek, Joanna A. Bartkowska, Lucjan Kozielski, Izabela Szafraniak-Wiza

**Affiliations:** 1 Institute of Materials Engineering, Faculty of Science and Technology, University of Silesia in Katowice, 75 Pułku Piechoty 1a, 41-500 Chorzów, Poland; joanna.bartkowska@us.edu.pl (J.A.B.); lucjan.kozielski@us.edu.pl (L.K.); 2 Institute of Materials Science and Engineering, Poznan University of Technology, Jana Pawła II 24, 61-138 Poznan, Poland; izabela.szafraniak-wiza@put.poznan.pl

**Keywords:** lead-free ceramics, mechanochemical activation, spark plasma sintering, negative dielectric constant, negative dielectric loss

## Abstract

This paper investigates the impact of the technological process (Mechanochemical Activation (MA) of the powder in combination with the Spark Plasma Sintering (SPS) method) on the final properties of lead-free Ba(Fe_1/2_Nb_1/2_)O_3_ (BFN) ceramic materials. The BFN powders were obtained for different MA duration times (x from 10 to 100 h). The mechanically activated BFN powders were used in the technological process of the BFN ceramics by the SPS method. The measurements of the BFN_x_MA ceramic samples included the following analysis: Scanning Electron Microscopy (SEM), Energy Dispersive Spectrometry (EDS), DC electrical conductivity, and dielectric properties. X-ray diffractions (XRD) tests showed the appearance of the perovskite phase of BFN powders after 10 h of milling time. The longer milling time (up 20 h) causes the amount of the perovskite phase to gradually increase, and the diffraction peaks are more clearly visible. Short high energy milling times favor a large heterogeneity of the grain shape and size. Increasing the MA milling time to 40 h significantly improves the microstructure of BFN ceramics sintered in the SPS technology. The microstructure becomes fine-grained with clearly visible grain boundaries and higher grain size uniformity. Temperature measurements of the BFN ceramics show a number of interesting dielectric properties, i.e., high values of electric permittivity, relaxation properties with a diffusion phase transition, as well as negative values of dielectric properties occurring at high temperatures. The high electric permittivity values predestines the BFN_x_MA materials for energy storage applications e.g., high energy density batteries, while the negative values of dielectric properties can be used for shield elements against the electromagnetic radiation.

## 1. Introduction

The multiferroic ceramic materials with the perovskite structure have been widely used in microelectronic and electronic applications and in a lot of fields of material engineering. Is well known that the best final piezoelectric properties have perovskite ceramic materials based on the solid solution PbZr_1−x_Ti_x_O_3_ (PZT) [1]. On the basis of this system, multi-component ceramic materials are designed to improve electrophysical properties as well as ceramic composite materials by introducing a magnetic factor into the chemical composition of the composite compound, e.g., ferrite [2], allowing extending the application possibilities of the material. Multiferroic materials show at the same phase two spontaneous orderings e.g., ferroelectric and magnetic [3]. There are many ferroelectromagnetic ceramic materials in this group, with interesting properties wherein lead-based materials also dominate, e.g., Pb(Fe_1/2_Nb_1/2_)O_3_ [4,5,6], Pb(Fe_2/3_W_1/3_)O_3_ [7], Pb(Fe_1/2_Ta_1/2_)O_3_ [8], or a combination of them [9]. Lead is connected to unfavorable technological factors—i.e., the formation of lead vacancies (during sintering at high temperatures) and the distorting stoichiometry or crystallographic structure of a compound—as well as harmful and adverse effects of lead on the environment and health human.

For many years, ceramic compounds and materials have been searched for that do not contain lead in their chemical composition, which could successfully replace ceramic materials with lead. These include BaTiO_3_, SrTiO_3_, BiFeO_3_, Bi_1/2_Na_1/2_TiO_3_, K_1/2_Na_1/2_NbO_3_, Na_1/2_Bi_1/2_TiO_3_, Na_1/2_Bi_1/2_TiO_3_, BaFe_1/2_Nb_1/2_O_3_, SrBi_2_Nb_2_O_9_, and a combination of them [10,11,12]. One of the lead-free materials widely studied in this direction is Ba(Fe_1/2_Nb_1/2_)O_3_ ceramics (BFN). BFN is a material with a perovskite structure and relaxor-type ferroelectric properties. BFN electroceramics exhibit attractive dielectric and electrical properties over a wide temperature range. At room temperature, the BFN has a monoclinic structure [13] or cubic structure with Fm-3m space group [14]. The characteristic features of the BFN are the diffuse phase transition and high values of electric permittivity but also high dielectric loss [15]. These high dielectric constants are influenced by both extrinsic as well as intrinsic factors [16]. In order to improve the dielectric properties of the BFN materials, different technologies for obtaining ceramics are used [17,18,19,20,21,22,23,24,25], or modifiers are introduced into the base compound [26,27,28,29].

High dielectric constant materials in modern applications are advantageous from the point of view of the miniaturization of electronic devices, i.e., in memory devices, power transmission, actuators, and sensors [30]. BFN material has excellent potential application in microelectronics, e.g., for capacitor applications [18] and energy storage applications (energy storage devices, high energy-density batteries) [22,26].

Usually, BaFe_1/2_Nb_1/2_O_3_ material is synthesized by the conventional mixed-oxide method, leading to coarse-grained powders [23] as well as wet-chemical synthesis routes, i.e., sol–gel and co-precipitate methods [21,22,25] or a microwave-assisted synthesis [18]. Another method of synthesizing is Mechanochemical Activation, which is defined as a process able to induce the structural disorder of the powders through intensive grinding. The Mechanochemical Activation process is extensively utilized in powder metallurgy, the synthesis of nanocomposites, or pharmacology. In the case of the polycrystalline BFN, nanomaterial has been successfully synthesized via the mechanochemical method in [19].

Recently, the Spark Plasma Sintering (SPS) method has been used in the sintering process of ceramic materials. SPS belongs to a class of powder metallurgy techniques using electric current and pressure for synthesizing materials in one step [31]. In the case of the conventional powder metallurgy methods, i.e., Free Sintering methods, Hot Pressing and Hot Isostatic Pressing Sintering long process times and high temperatures are used. SPS methods of sintering progress quickly because the heat is generated by the electrothermal effect and distributed only within the sintering area [31]. The advantages of the SPS method over conventional sintering techniques are summarized as follows: higher heating rate, lower sintering temperature, and shorter holding time. In most materials with a perovskite structure, the use of the SPS method improves the electrophysical parameters of ceramic samples.

An aim of the work was to obtain the Ba(Fe_1/2_Nb_1/2_)O_3_ (BFN) ceramics using the combined technological process i.e., Mechanochemical Activation (MA) of the ceramic powders (with different mixing times) and sintering by Spark Plasma Sintering (SPS) method and the influence of a complex technological process on final physical properties. So far, no attempt has been made to combine both above-mentioned technological methods in the technological process to obtain BFN ceramic materials. The use of the SPS sintering method should have a positive effect on the electrophysical properties of BFN ceramics.

## 2. Experimental

### 2.1. Materials and Methods

BFN ceramic powder was prepared by the high-energy milling method (Mechanochemical Activation). As input components, the high-purity powders, i.e., iron oxide Fe_2_O_3_ (>99%, Sigma-Aldrich, St. Louis, MO, USA), niobium oxide Nb_2_O_5_ (99.9%, Sigma-Aldrich, St. Louis, MO, USA), and barium carbonate BaCO_3_ (99.99%, POCH, Gliwice, Poland) have been used, and they are connected to each other according to the reaction: BaCO_3_ +0.25Fe_2_O_3_ +0.25Nb_2_O_5_ → BaFe_1/2_Nb_1/2_O_3_ +CO_2_↑. The powders were pre-homogenized in a planetary ball mill for 8 h (in ethyl alcohol), and next, the dried mixture was mechanically activated by the ball-milling method using an SPEX 8000 Mixer Mill (SPEX SamplePrep, Metuchen, NJ, USA) with stainless steel balls (in the air atmosphere). In the MA method, the ball/powder weight ratio was 5/1. In this process step, we applied a different period powder milling, i.e., 10, 20, 40, 60, 75, and 100 h.

In the next step of the technological process, pre-synthesized BFN powders (by MA method) were sintered by the Spark Plasma Sintering (SPS) method. All BFN ceramic samples were carried out in the following conditions: temperature—1000°C, time—3 min., pressure—50 MPa, atmosphere—argon, heating rate—50 °C/min. The ceramic samples are named as BFN_x_MA where x means the duration of high energy milling time, i.e., BFN_10_MA (10 h), BFN_20_MA (20 h), BFN_40_MA (40 h), BFN_60_MA (60 h), BFN_75_MA (75 h), and BFN_100_MA (100 h).

### 2.2. Characterization

The milling process (at various times of the MA) was controlled by X-ray diffraction studies by a PANalytical Empyrean X-ray powder diffractometer (PANalytical B.V., Almelo, The Netherlands) with CuK_α_ radiation (45 kV, 40 mA) at room temperature (RT). The surface morphology of ceramic samples (SEM microstructures) and EDS (Energy-Dispersive Spectrometry) tests were made by a scanning electron microscopy JSM-7100F TTL LV (Jeol Ltd., Tokyo, Japan). The surfaces of tested samples were coated with a layer of gold to provide electrical conductivity, in order to avoid charging effects.

Dielectric measurements were performed using a capacitive bridge of a QuadTech 1920 Precision LCR Meter (Quad/Tech, Inc., Maynard, MA, USA) during the heating cycle (from RT to 500 °C) in the frequency range from 1 kHz to 1 MHz. The measurements of direct current electrical conductivity were performed using a Digital Multimeter Device USB-4065 (National Instrumental, Austin, TX, USA) in the temperature range from RT to 530 °C.

## 3. Results and Discussion

**Figure 1:**
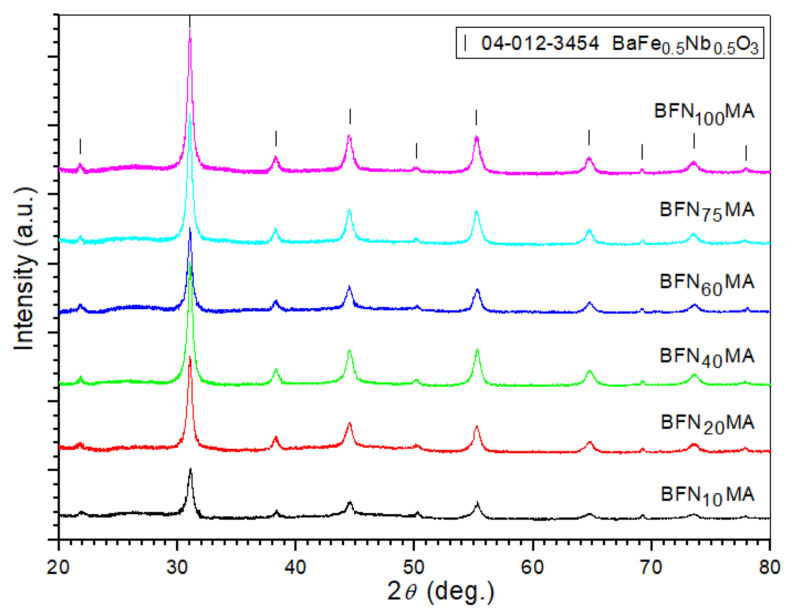
X-ray diffraction (XRD) patterns of the BaFe_1/2_Nb_1/2_O_3_ (BFN) powders after different periods of high-energy milling.

For the synthesis control, during the high-energy milling process, the ceramic powders were examined by X-ray diffraction (XRD) measurements after each milling time period, i.e., 10, 20, 40, 60, 75, and 100 h (Figure 1). The synthesis process has triggered already after starting 10 h of the high-energy milling duration and the diffraction patterns caused the gradual appearance of the crystallographic structure of BFN material. Initially, the detected perovskite peaks are weak, but longer milling up 20 h causes the amount of the perovskite phase to gradually increase, and the diffraction peaks become more sharp and clearly visible (their intensities increased, too). The XRD tests have revealed several clear peaks assigned to the perovskite phase, but the relatively considerable background of the diffraction patterns may indicate the presence of an amorphous phase. In addition, for the longer milling periods observed, the peaks become broader, which suggests a significant refinement in the crystalline size. XRD tests of the BFN powders show no significant difference for longer milling time. The best fit to the experimental results was obtained with the BaFe_1/2_Nb_1/2_O_3_ pattern (no. card 04-012-3454) belonging to the cubic system (space group Pm-3m).

**Figure 2:**
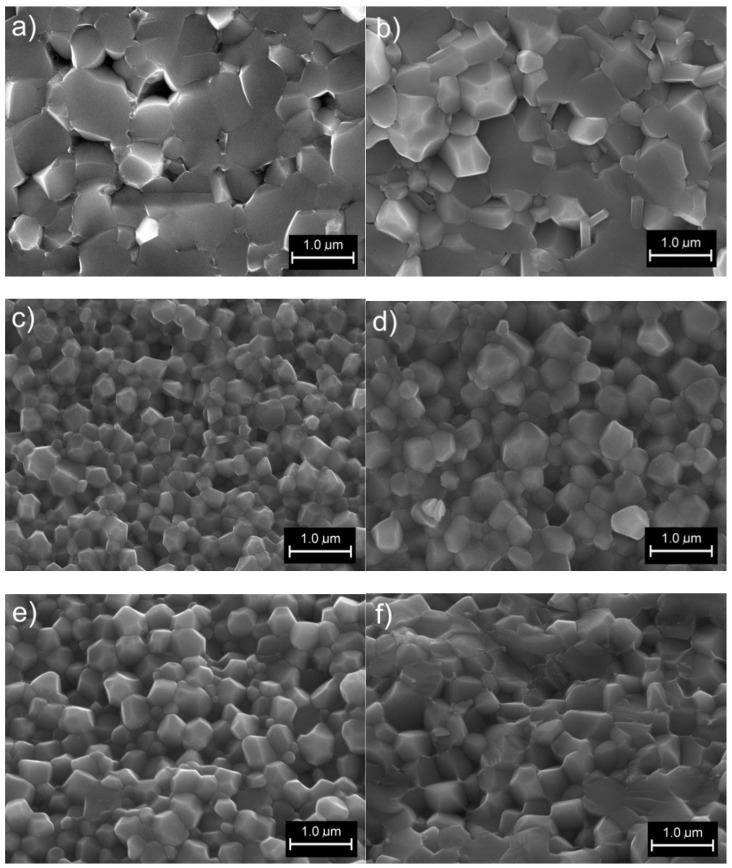
Scanning Electron Microscopy (SEM) images of the microstructure of fracture of the BFN_x_MA ceramic samples: BFN_10_MA (**a**), BFN_20_MA (**b**), BFN_40_MA (**c**), BFN_60_MA (**d**), BFN_75_MA (**e**), and BFN_100_MA (**f**) (magnification ×20,000).

Figure 2 presents a summary of the fracture surface morphology of BFN samples obtained at different times of high-energy milling and sintered by the SPS method. In the case of short mixing times, a large heterogeneity of the grain shape and size is observed. Poorly comminuted powder grains promote excessive grain growth. For these samples, the grain growth during the SPS process is heterogeneous; i.e., the poorly fragmented grains (larger grains) grow much faster than the finer grains, creating a heterogeneous microstructure (Figure 2a,b). The fractures of the samples are both at the grain boundary and through the grains. Increasing the high-energy milling time to 40 h significantly improves the microstructure of BFN ceramics sintered in the SPS technology. The microstructure becomes fine-grained with clearly visible grain boundaries and increased grain size uniformity. The fine ceramic grains are characterized by the correct shape, and the fracture occurs along the grain boundary. This means that the mechanical stresses resulting from the SPS sintering process are mainly deposited at the grain boundary, and the grains have a strong and with durable structure. The optimum fine-grained microstructure is shown already for the BFN_40_MA sample. In the case of the BFN_100_MA composition, the grain size heterogeneity increases, and the breakage also occurs through the grain. The reason for this may be a significant increase in mechanical stress and the defects of the grains produced during long-term high-energy milling.

The fine-grained microstructure of ceramic samples obtained by the complex technology (MA and SPS) shows the appearance characteristic of ceramic materials obtained by the sol–gel method [32], and it is completely different from the microstructure of BFN ceramics obtained by the free sintering method, which requires high sintering temperatures [33] as well as MA and the free sintering method [34].

**Figure 3:**
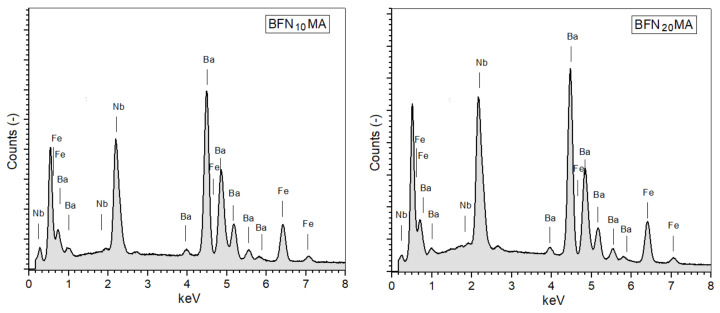

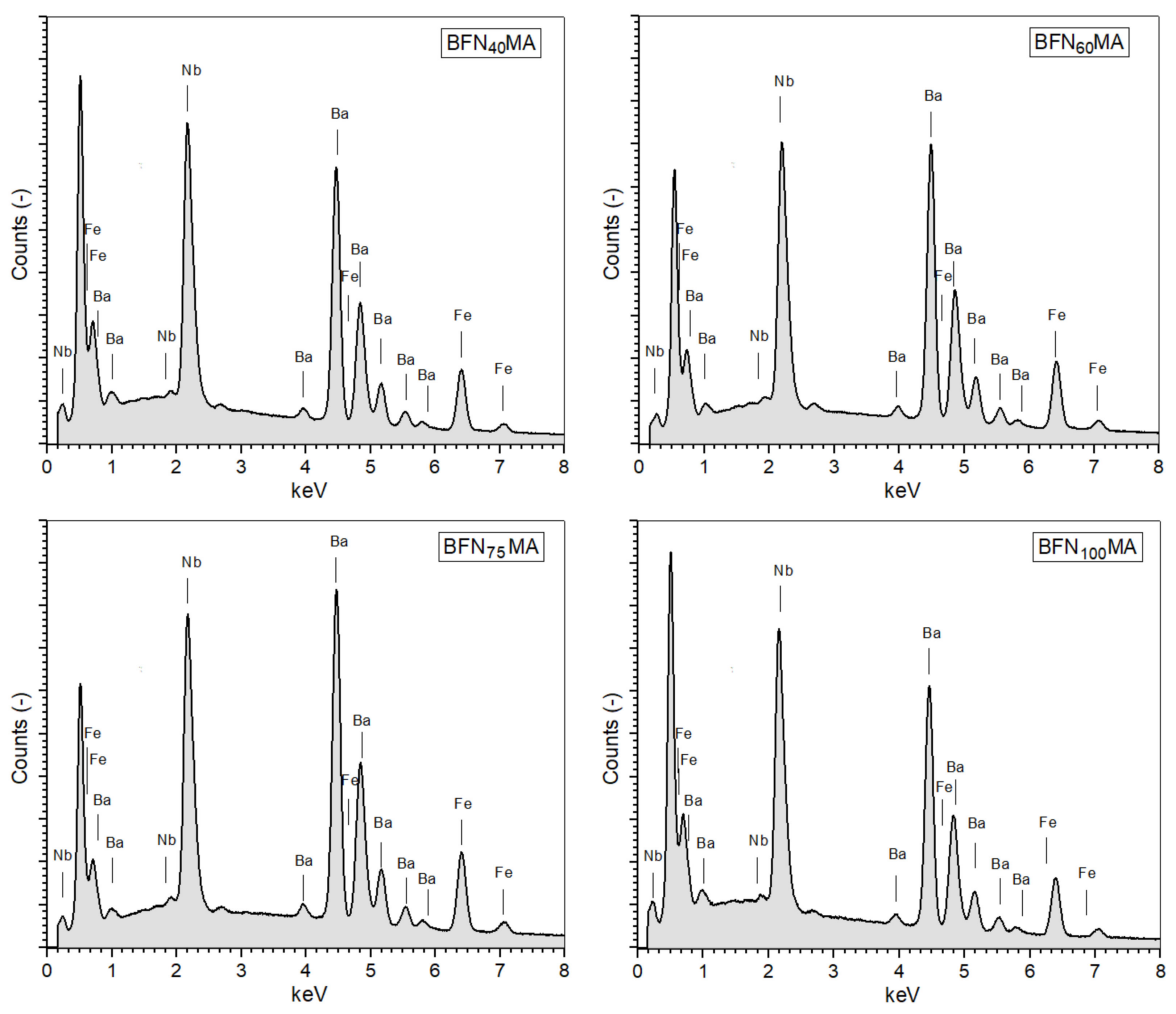
The Energy Dispersive Spectrometry (EDS) analysis of chemical elements of the BFN_x_MA ceramic samples: BFN_10_MA, BFN_20_MA, BFN_40_MA, BFN_60_MA, BFN_75_MA, and BFN_100_MA.

The qualitative energy dispersive spectroscopy (EDS) analysis was used to investigate the chemical composition of the BFN ceramic samples and their results are presented in Figure 3. The microanalysis was performed at micro-areas on the fractures of samples (the results are the average value of five randomly selected sample areas). The qualitative EDS investigations confirmed the assumed share of the individual components, without foreign elements and impurities. The results have shown that duration of high-energy milling affects the correct grinding of the powder, which contributes to the occurrence of local differences in the chemical composition in the volume of the ceramic sample. Overall, the EDS study showed a slight increase in the amount of barium and iron while decreasing the amount of niobium and oxygen in the ceramic samples in comparison with theoretical calculation (Table 1). However, the chemical composition deviation is in an acceptable range for BFN ceramics.

The research presented in the literature shows that the BFN ceramic material has high DC electrical conductivity [17,19]. At room temperature, the resistivity values of the ceramic samples take average values, i.e.,: 1.29 × 10^5^ Ωm (for BFN_10_MA), 9.35 × 10^5^ Ωm (for BFN_20_MA), 2.29 × 10^6^ Ωm (for BFN_40_MA), 1.67 × 10^6^ Ωm (for BFN_60_MA), 3.26 × 10^6^ Ωm (for BFN_75_MA), and 4.89 × 10^4^ Ωm (for BFN_100_MA). At higher temperatures, the increase in electrical conductivity in the BFN_x_MA materials is significant.

**Figure 4:**
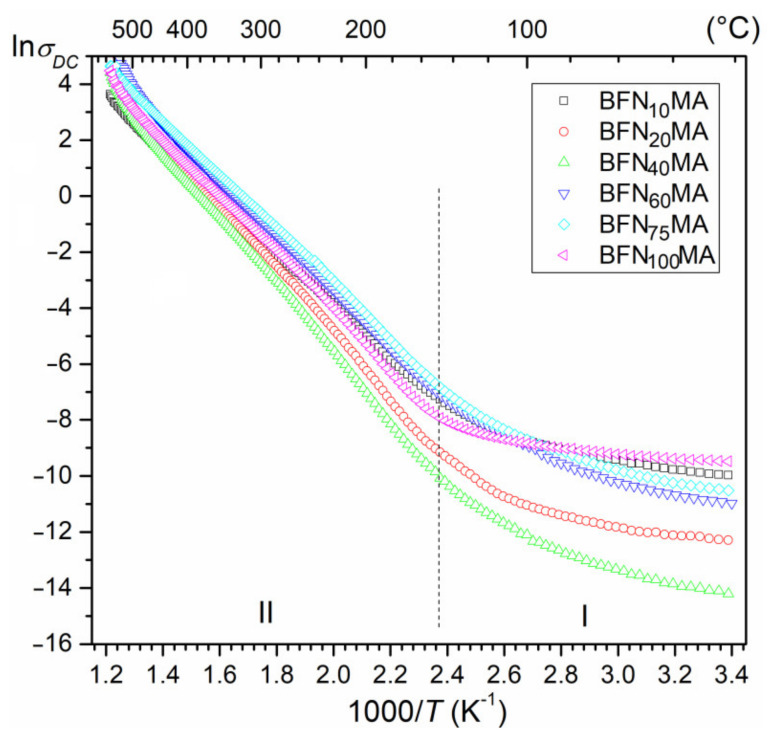
Temperature dependencies of electric conductivity for BFN_x_MA ceramic samples.

Figure 4 shows the dependence of the lnσ_DC_ on reciprocal of temperature (1000/T) for a series of BFN_x_MA samples. The lowest direct current electrical conductivity was observed for the BFN_40_MA sample, while at higher temperatures, the nature of changes in the lnσ_DC_ (1000/T) curves is similar for individual samples. Based on the slope of the curves lnσ_DC_ (1000/T) and Arrhenius’ Equation (1), the activation energies E_a_ were calculated. The values of electric conductivity and the calculated activation energies in two temperature areas, i.e., ferroelectric phase (T < T_m_) and paraelectric phase (T > T_m_) in the distinctive areas are presented in Table 2.

(1) $$\sigma_{\mathrm{DC}}=\sigma_{0}\exp\left(\frac{E_{a}}{k_{B}T}\right).$$

where σ_0_—pre-exponential factor, k_B_—Boltzmann’s constant, T—absolute temperature, and E_a_—the activation energy. The E_a_ values of the BFN_x_MA ceramics (at higher temperatures) have been found in range from 0.79 to 1.0 eV (Table 2). Activation energy value usually means the relaxations, which include the movement of cations due to the applied energy as well as the thermal motion of anions. The long-range hopping of oxygen ions between neighboring sites can be a possible cause of this type of relaxation [19]. Similar activation energy values of the BFN ceramics made by the SPS method were obtained in [17].

**Figure 5:**
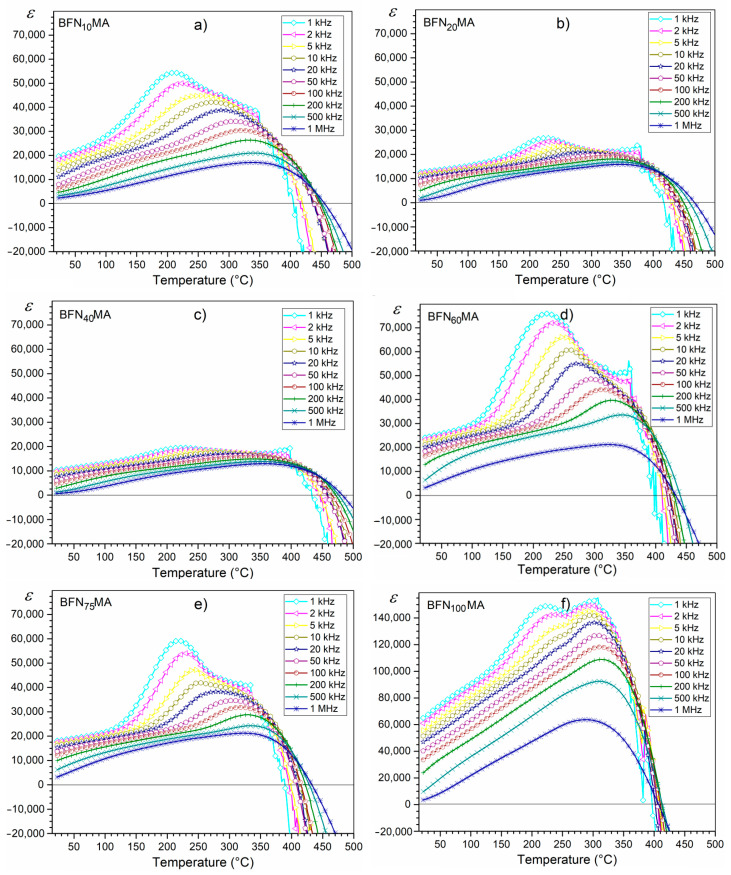
Temperature dependencies of electric permittivity for BFN_x_MA ceramic samples: BFN_10_MA (**a**), BFN_20_MA (**b**), BFN_40_MA (**c**), BFN_60_MA (**d**), BFN_75_MA (**e**), and BFN_100_MA (**f**).

Lead-free BFN ceramics obtained in the combined technological process (high-energy milling and Spark Plasma Sintering) are characterized by a number of interesting dielectric properties, which include, among others, high values of electric permittivity, relaxation properties with a diffusion phase transition as well as negative values of electric permittivity and dielectric loss occurring at high temperatures. The maximum electric permittivity occurs in the area of the phase transition, which is characteristic of ceramic materials with a perovskite structure. In the case of BFN ceramics, the phase transition from the ferroelectric to paraelectric phase is strongly blurred. The maximum T_m_ temperature appears around 220 °C. The exception is sample BFN_100_MA (with the longest milling time), where a second maximum electric permittivity appears too, at the higher temperature. The ε(T) curves show typical relaxor behavior of ceramic material (Figure 5). The peak maximum of electric permittivity is shifting toward higher temperature, while the magnitude of the ε is decreasing with the increasing frequency field. This accompanied by a wide diffused phase transition. Similar results are presented by Majumder et al. [4], and Ganguly et al. [23] explained it as a phenomenon associated with disorder in the B-site cation distribution occurring in A(B’B”)O_3_ type perovskite materials. The random distribution of ions in the B positions of the compound contributes to the presence in the ceramic sample of micro-areas with different local Curie temperature, which causes the ferroelectric–paraelectric phase transition in a wide range of temperatures. In the case of the BFN_100_MA sample, the phenomena are higher compared to those of the other BFN_x_MA samples.

**Figure 6:**
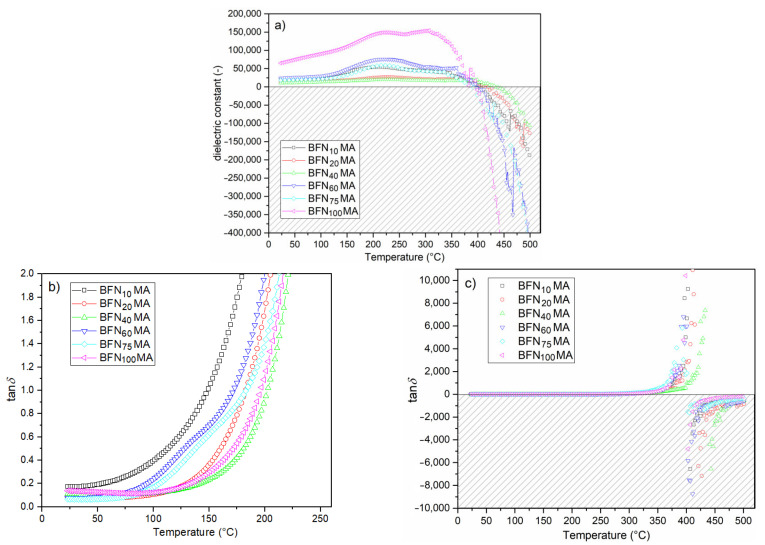
Temperature dependencies of dielectric properties for the BFN_x_MA ceramics (1 kHz): electric permittivity (**a**), dielectric loss (from RT to 250 °C) (**b**), and dielectric loss (from RT to 500 °C) (**c**).

BFN_x_MA ceramic samples show high values of electric permittivity. At room temperature, they range from 10,330 to 64,510, while at T_m_, they range from 19,520 to 148,920 (for 1 kHz—Figure 6a). These high values of electric permittivity are the result of external and internal factors. Intatha et al. [35] claim that the high value of ε is associated with a heterogeneous conduction in the grain and grain boundary. Such a phenomenon occurs due to the significant separation of grains by the wider insulating intergrain barrier. It is also well known that during technological processes using high sintering temperatures, oxygen vacancies and Fe^2+^ are created, resulting in growth of the electrical conductivity, dielectric loss, as well space charge, an excess of which accumulates on the grain boundaries [16]. The ceramic samples obtained by a combined method (by MA and SPS techniques) show very higher values of electric permittivity at room temperature in comparison with BFN ceramics obtained by the Solid-State Reaction Method [23].

BFN ceramics is an interesting material in terms of the possibility of new applications (e.g., as a shield against electromagnetic radiation) due to its negative dielectric properties at higher temperatures (Figure 5 and Figure 6a). Above the phase transition temperature, the electric permittivity values systematically decrease, and the ceramic BFN samples show negative dielectric constant values above 420 °C (depending on the frequency of the field) for all frequencies of measuring the electric field (Figure 5). The first reports of such rare dielectric behavior in the real material were presented Mao et al. [36,37]. In recent years, more and more papers present the occurrence of this phenomenon in various ceramic materials [38,39,40], but so far, the mechanism of the appearance of a negative dielectric constant is not yet fully examined and explained. Mao et al. [36] reported that the negative value of the dielectric constant is related to the change in the nature of the electrical conductivity from capacitive to inductive. The negative value of the dielectric constant can be interpreted by analogy with magnetic materials. Namely, the negative value of magnetic susceptibility proves that the material exhibits magnetization opposite to the field (diamagnetism). So, the negative value of the dielectric constant may indicate that the dipoles are positioned opposite to the external electric field. The electric field formed inside the ceramic material under such conditions will weaken the external electric field impact (causing its shielding) [38]. Thus, such material behaves similar to a diaelectric—namely, it is pushed out of the external electric field [39,41,42].

Another approach to describe the negative value of the dielectric constant can be relaxation phenomena, assuming that during the relaxation process, the number of holes in the material increases. These added holes can recombine with free electrons in the dipoles. The phenomenon of such recombination causes a decrease in the charges of the dipoles, which causes the appearance of a negative value of the dielectric constant [43]. The possibility of shielding the external electric field by a material with a negative dielectric constant value is important for e.g., optoelectronic application [42]. Materials characterized by such rare properties, i.e., the negative dielectric constant, can be called a diaelectric material [40].

**Figure 7:**
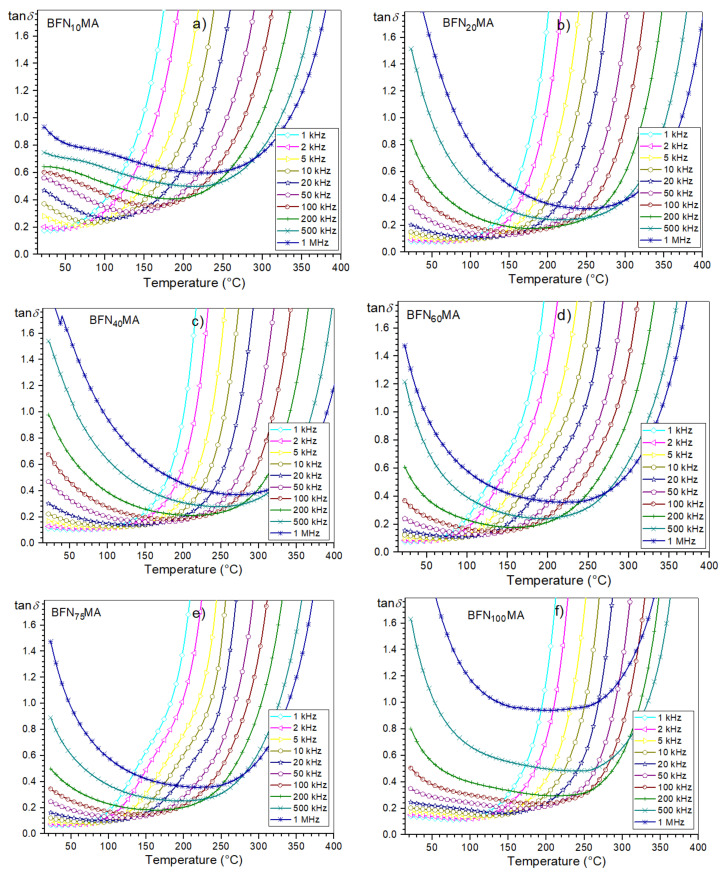
Temperature dependencies of dielectric loss for BFN_x_MA ceramic samples: BFN_10_MA (**a**), BFN_20_MA (**b**), BFN_40_MA (**c**), BFN_60_MA (**d**), BFN_75_MA (**e**), and BFN_100_MA (**f**).

**Figure 8:**
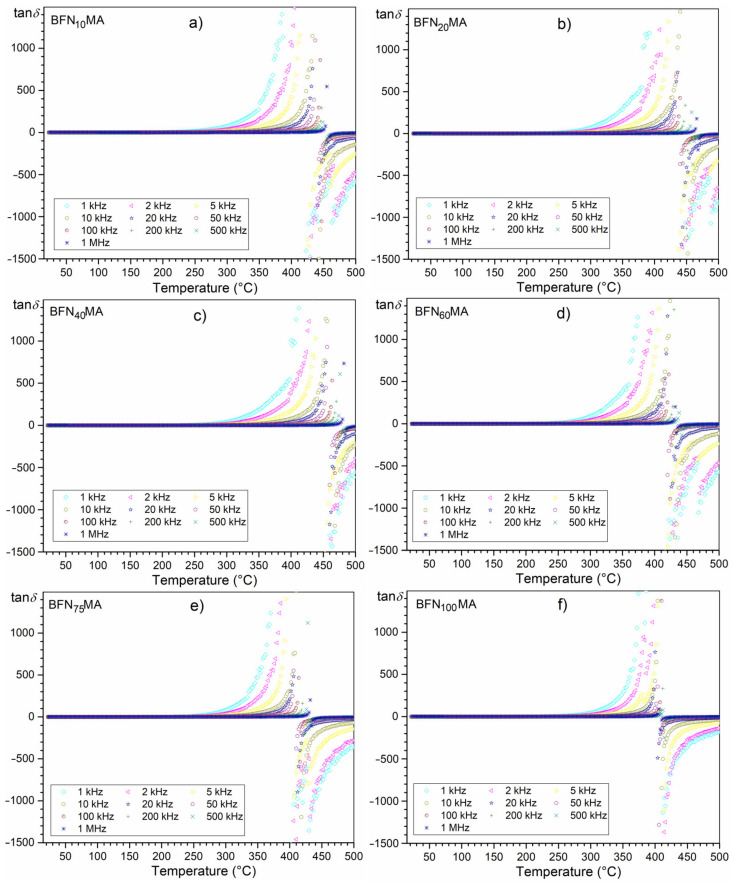
Temperature dependencies of dielectric loss for BFN_x_MA ceramic samples: BFN_10_MA (**a**), BFN_20_MA (**b**), BFN_40_MA (**c**), BFN_60_MA (**d**), BFN_75_MA (**e**), and BFN_100_MA (**f**) in a wider measuring range.

Temperature dependencies of the dielectric loss for 1 kHz in a narrow temperature range (from RT to 250 °C) for all tested samples are shown in Figure 6b. At room temperature (for 1 kHz), the values of dielectric loss of the ceramic samples are relatively low i.e.,: 0.17 (for BFN_10_MA), 0.08 (for BFN_20_MA), 0.11 (for BFN_40_MA), 0.08 (for BFN_60_MA), 0.07 (for BFN_75_MA), and 0.14 (for BFN_100_MA). Figure 7 presents the temperature dependencies of dielectric loss tangent (tanδ) for all BFN ceramic samples in the range from RT to 400 °C. It can be observed that initially, the dielectric loss values decrease to some positive values and then increase for all frequencies of the electric measurement field. All BFN ceramic samples show similar character of dielectric loss at higher temperatures. The dielectric loss depends on the frequency of the electric measurement field. At room temperature, higher frequency results at higher dielectric loss, while at higher temperatures, the opposite occurs (at higher frequency, the lower the dielectric loss is observed). As is commonly known, in the case of the materials with a perovskite structure, the dielectric loss depend on many factors, i.e., the structural homogeneity, stoichiometry, the amount of free electric charge carriers, the chemical composition of the sample, and technological process conditions. At a certain temperature, i.e., above 420 °C (depending on the frequency of the field), the dielectric loss tangent reaches its maximum value, and then its value drops sharply with further temperature increase (Figure 8). The decrease in the value of the dielectric loss tangent goes down to negative values. It can be observed that the values of the tanδ depend inversely on the values of frequency of the measurement field; i.e., the higher the frequency, the lower the dielectric loss. The behavior takes a characteristic appearance (for limit of the function) with a vertical asymptote (which is located at different temperatures depending on the frequency) and a horizontal asymptote (at 0). This behavior of the dielectric loss tangent can be caused by the domain walls resonance [44].

The positive values of dielectric loss are the result the normal relaxation processes; e.g., they may be related to the presence in the ceramic material of both Fe^3+^ and Fe^2+^ ions. Iron Fe^2+^ ions can be created as a result of a reduction of Fe^3+^ ions during sintering at high temperature [45]. The created electric dipoles cause the polarization of material, but there is certain inertia during rotation of the dipoles, which results in the existence of a relaxation process. The temperature dependencies of the dielectric loss describe the processes that are related to the absorption and emission of energy. Axelrod et al. were the first to become interested this phenomenon in 2006 and called it the Negative Losses Process (NLP) [46]. The negative dielectric loss may indicate that more energy is emitted than absorbed in the material. In order to fulfill the principle of conservation of energy in the case of materials with negative dielectric loss, it must be assumed that the absorbed energy is stored in the material together with the trapped electrons (e.g., on the surface of the pores). However, such a phenomenon is not stable, and therefore, under certain specific conditions, such as temperature or frequency, the stored energy is released in an avalanche nature. Such avalanche emission of energy manifests itself in the negative value of dielectric loss [47].

The occurrence of the negative dielectric loss (Figure 6c and Figure 8) can mean the presence in real material of the local non-compensated charges (real material always exhibits some internal porosity). These non-compensated charges accumulate easily on the interface of the pores. The generated charges may be also anchored via non-bonding orbitals that are full or empty for positive or negative ions, respectively. This assumption allows explaining the mechanism of separation of the space charges. The separation of charges leads to a metastable state in material with the accumulation of energy [48]. The presence of the metastable state may be changed under certain conditions, e.g., using the appropriate temperature and frequency. A suitable temperature and frequency can initiate a recombination of charges, which results in the effect of energy emission [48].

## 4. Conclusions

The lead-free Ba(Fe_1/2_Nb_1/2_)O_3_ (BFN) ceramic samples made by the combined method, i.e., Mechanochemical Activation and Spark Plasma Sintering, were successfully obtained and examined. The BFN powders were synthesized via MA for different duration times between 10 and 100 h, while the BFN_x_MA ceramic samples were sintered using the SPS method.

XRD tests showed the appearance of the perovskite phase of BFN powders after 10 h of milling. The SEM images show that increasing the MA milling time to 40 h significantly improves the microstructure of BFN_x_MA ceramics sintered by the SPS method. The microstructure becomes fine-grained with clearly visible grain boundaries and higher grain size uniformity. The EDS tests confirmed the proper chemical composition of BFN_x_MA ceramic samples without foreign elements and impurities. At RT, the resistivity values of the BFN_x_MA ceramic samples take average values, i.e., ranging from 4.89 × 10^4^ Ωm to 3.26 × 10^6^ Ωm.

Temperature dielectric measurements of the lead-free BFN_x_MA ceramics show a number of interesting properties, i.e., high values of electric permittivity, relaxation behavior with a diffusion phase transition, as well as a negative values of electric permittivity and dielectric loss occurring at high temperatures (above 400 °C). The reason for the negative dielectric constant value may be the change of the type of conductivity from capacitive to inductive. The negative values of the dielectric loss mean that energy is absorbed and stored in material, whereas it is emitted in an avalanche way under appropriate conditions (i.e., suitable temperature, frequency). Due to the fact that the phenomenon of negative dielectric properties occurring in ceramic perovskite materials is not fully understood and explained, further analyzes and additional investigations are required.

The high electric permittivity values, both at room temperature and at Curie temperature predestines the BFN_x_MA materials for energy storage applications, e.g., high-energy density batteries, whereas the negative values of dielectric properties can be used for shield elements against the electromagnetic radiation.

## Figures and Tables

**Table 1:** Theoretical and experimental percentage of the individual components of the BFN_x_MA ceramics.

Elements	Theoretical (Mass %)	BFN_10_MA	BFN_20_MA	BFN_40_MA	BFN_60_MA	BFN_75_MA	BFN_100_MA
		Experimental Data (mass %)					
Ba	52.879	54.3	53.72	53.2	53.05	53.12	53.36
Fe	10.752	11.36	11.17	11.04	11.56	11.65	11.08
Nb	17.887	16.87	17.64	17.84	17.75	17.65	17.83
O	18.482	17.47	17.47	17.92	17.64	17.58	17.73

**Table 2:** Electrophysical properties of the BFN_x_MA materials.

Parametr	BFN_10_MA	BFN_20_MA	BFN_40_MA	BFN_60_MA	BFN_75_MA	BFN_100_MA
T_m_ (°C)	210	224	221	221	220	220/298
ε_p_	19,678	12,610	10,330	24,120	18,080	65,010
ε_m_	54,450	26,900	19,710	75,900	59,240	148,920/152,920
tanδ at RT	0.17	0.08	0.11	0.08	0.07	0.14
tanδ at T_m_	4.81	3.96	1.99	3.90	2.61	2.37/43
E_a_ at I (eV)	0.13	0.16	0.28	0.21	0.22	0.09
E_a_ at II (eV)	0.79	0.95	1.00	0.85	0.83	0.88

## Data Availability

Data is contained within the article.
